# Eligibility for the kidney transplant wait list: a model for conceptualizing patient risk

**DOI:** 10.1186/2047-1440-3-2

**Published:** 2014-01-08

**Authors:** Bryce A Kiberd, Karthik K Tennankore, Kenneth West

**Affiliations:** 1 Division of Nephrology, Department of Medicine, Dalhousie University, Room 5082 Dickson Building, Queen Elizabeth II HSC-VG site, 5280 University Avenue, Halifax B3H 1V8, NS, Canada

**Keywords:** Mortality, Wait list, Frailty, Access, Allocation, Kidney transplantation

## Abstract

**Background:**

Determining eligibility for a kidney transplant is one of the most important decisions facing nephrologists. It is assumed that the harm of kidney transplantation is minimal and most will benefit. The purpose of this study was to quantify the probability of ‘no benefit’ as defined by death on the wait list; ‘harm’, defined by the probability that a transplanted patient would live less than the average wait listed patient; and ‘benefit’ for the probability a transplanted patient would outlive the average wait listed patient.

**Methods:**

A computerized model was developed to replicate observed patient survival outcomes in deceased donor kidney transplantation. Three sequential periods of risk for the transplanted recipient compared to the wait listed cohort (increased, equivalent and reduced risk) were modeled.

**Results:**

The model predicted that wait listed patients with a baseline mortality of 28 deaths per 100 patient years were equally likely to benefit or be harmed with a transplant. However if 20% of patients on the wait list were on hold (assuming a 2.2-fold higher mortality than those who were transplanted), then the baseline mortality rate for equal harm or benefit decreases to 22 deaths per 100 patient years (equivalent life expectancy 4.5 years).

**Conclusion:**

Patients with limited life expectancies are more likely to suffer some harm than derive benefit from kidney transplantation.

## Background

Much has been written about the principles of allocating deceased kidney organs to those on the wait list. Less has been written about ethical principles of wait list eligibility. There are several principles that seem reasonable, namely, exclusion of patients who do not want a transplant (autonomy), exclusion of patients where the operation or immunosuppression are likely to cause greater harm (non-maleficence), and exclusion of patients not likely to benefit in deference to those who are likely to benefit (utilitarianism). Some suggest that patients with a life expectancy of less than five years should not be considered for transplantation as they are not likely to derive significant benefit [1]. A Canadian recommendation is not to evaluate individuals who are unlikely to survive the wait period [2]. Some would argue that anyone who might benefit regardless of the risk should have equal access to kidney transplantation. Most agree that excluding transplantation based solely on age is unjust [3].

There is considerable variation in the wait list as a percentage of those on dialysis between countries, within countries and within regions [4]. There no clear explanation of this variation and many believe that eligibility for listing is not transparent and worthy of further evaluation [5]. On a patient level, being able to predict who is likely to be harmed and who is likely to benefit is unclear. Some of the confusion has been generated by the belief that since all patient groups that are transplanted have a net benefit in life years gained (even for those aged 70+), transplantation is being denied to many that would benefit [6,7].

A detailed analysis of the patient survival demonstrates that there is an increased risk of early death among transplanted patients compared to those remaining on the wait list [7,8]. The magnitude and duration of increased risk may vary by organ source (live versus deceased), recipient age and organ quality. Generally, the period of increased risk is < six months. Since most patients survive the first six months, transplantation appears to benefit most.

However there are several caveats that bear scrutiny. Many on the transplant wait list are on hold and these patients are not transplanted. Those on hold/inactive have recently been shown to have a 2.2-fold greater risk of death than those who are active [9]. Since earlier studies did not adjust for this, the net benefit has been significantly overestimated. In addition, high wait list mortality coupled with long wait times means that a significant proportion will die on the wait list [10]. For those that die or are removed from the list, there will be needless expense and intrusiveness of evaluation and re-testing annually without benefit. Lastly, even though the period of increased risk is relatively short (< six months), the time to equal percent survival (intersection of the two survival curves), and equal cumulative life years is progressively longer. For example, in elderly patients aged 70+, the period to equal risk was 125 days, but the time to equivalent percent survival was > 1.5 years [7]. No study has calculated the time to equivalent life years (area under the curves), which likely occurs > 2.5 years post transplantation in patients with higher mortality rates. Theoretically, patients who are on the list can have no benefit (die on the list), be harmed (receive a transplant but die before those who were never transplanted), or benefit (receive a transplant and outlive wait listed patients). It is mathematically possible to derive the likelihood an individual is to terminate in one of these outcomes (‘no benefit', ‘harm', and ‘benefit’) based on their inherent mortality rate. The purpose of this study is to examine how likely it is that a patient will be harmed compared to benefit assuming all patients expose themselves to an initial increased risk with transplantation. Rather than calculate a net benefit in life years, the premise is that there will be a mortality rate, above which, those who are transplanted are more likely to be harmed than to benefit on a proportion basis. Arguably, individuals with such a high mortality rate should not proceed to transplantation.

## Methods

Previous observational studies which have examined age stratified cohorts of patients on the wait list have calculated the net benefit of transplantation by comparing survival of those transplanted to those who remained on the list. The analysis assumed that those transplanted and those remaining on the wait list were equivalent patients. The calculation of net benefit assumed that that the comparator group (versus the transplanted group) remained on the wait list until death [6-8]. Patients put on hold were not transplanted but remained in the wait listed cohort and those that were removed from the list for illness were censored. The study by Rao *et al*., specifically examined an older cohorts of patients aged 70+ years [7]. In their study, the four year survival was 51% for the wait list cohort and 66% for the transplant cohort; however, the survival for the transplant cohort was inferior to the wait list cohort up until 1.5 to 2 years post follow up. To describe this phenomenon, they demonstrate an initial period of increased mortality risk that gradually tapers to a reduced relative mortality for the transplant cohort over time relative to the non-transplant cohort. It is not clear from their methods, but they likely assumed mortality rates were stable in their long-term calculation of net benefit.

**Figure 1:**
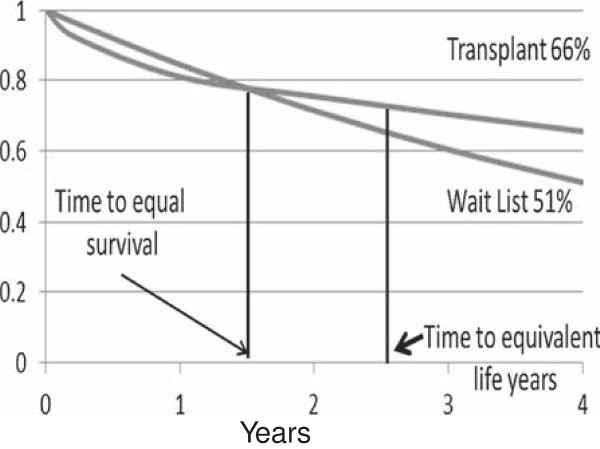
Modeled survival of transplant compared to wait list cohorts.

For this paper’s model, we assume there are three periods of relative risk: a period of increased risk (for the first 0.2 years), a period of equal risk for the remaining first year, and a period of reduced mortality risk for the transplanted cohort relative to the wait list group after one year. Relative risks were generated to model actual survival with time to equal percentage survival between 1.5 to 2 years, and overall survival of 66% for transplanted patients and 51% survival for those on the list at year 4 (Figure 1) as demonstrated by the actual survival for the cohort described by Rao *et al*. [7]. The survival model assumed a simple exponential decline and mortality rates (MR) in deaths per 100 patient years were converted to a survival probability:

(exp^(−MR*t)), where t is equal to time in years.

We found that assuming an increased risk period of 0.2 years with a relative risk of 2.26, followed by 0.8 years of equivalent risk and then a reduced risk of 0.44 produced a four year survival of 66% for the transplanted cohort and 51% for the wait list cohort, with a time to equal percent survival of 1.6 years.

**Figure 2:**
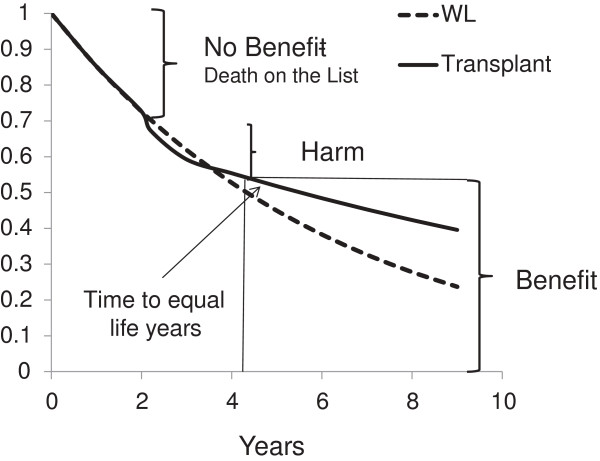
Graphic description of ‘no benefit’, ‘benefit’ and ‘harm’.

To explore other cohorts, we did not assume that patients were of a certain age but rather examined theoretical cohorts with similar mortality rates and compared survival assuming a group was transplanted and a group remained on the wait list until death. This initial analysis assumes all are equivalent candidates, none are on hold and there are no drop outs. A range of mortality rates (deaths per 100 patient years) was then examined in increments of five starting at 15 and progressing to 35 deaths per 100 patient years. We assumed that mortality rates were fixed over time for individuals with the same baseline mortality rate. For each death rate, the time to equal cumulative life years was calculated as the time at which the area under the survival curves for the wait list cohort and transplant cohort was equal (Figure 1). This time was calculated by integrating the area under the survival curves (see Additional file 1). The percent survival in the transplanted group at the time of equal cumulative life years was used to determine the transition from ‘harm’ to ‘benefit’. To simplify the baseline model, we assumed that all patients waited exactly two years on the list. In addition, analyses with three and four years on the wait list were performed. Figure 2 shows visually how the percentages for ‘no benefit', ‘harm’ and ‘benefit’ were derived.

Unfortunately, some patients on the wait list are on hold and these have recently been shown to have 2.2-fold higher mortality rates [9]. Since hold patients are not transplanted but are averaged in with those that are eligible, those that are transplanted have a proportionately lower mortality rate. By including non-transplantable patients in the wait list cohort, previous estimates of the net benefit of transplantation are overestimates. In an additional sensitivity analysis, wait list inactivity was incorporated into the model. Adjustments were made to assume that 10% or 20% of the wait list were ineligible due to inactive status and the reference wait list mortality was thereby reduced. This adjustment results in an increase in the mortality hazard ratio during the increased risk period and reduces the net benefit in the long term. For example, if 20% of the cohort is on hold, the true relative risk during the increased mortality risk period is 2.8 (versus 2.26) and the period of reduced relative mortality risk is 0.55 (versus 0.44) for those transplanted relative to the wait list cohort. Given the relatively small confidence interval for the increase risk of wait list mortality for those on hold (2.21, 95% confidence interval, 2.15, 2.28), an additional sensitivity analysis on the magnitude of this risk was not performed [9].

Another way of reporting survival is to estimate life expectancy rather than mortality rates. Based on our assumption of exponential decline in survival, life expectancy (area under the survival curve) is equal to 1/MR [10].

## Results

Patients with higher mortality rates show an increasing likelihood of ‘no benefit’ and ‘harm’ and diminishing likelihood of ‘benefit’ (Table 1). Assuming a mortality rate of 35 deaths per 100 patient years, transplantation is slightly more likely to harm (29%) than benefit (21%), whereas the remaining 50% of the patients are predicted to die on the wait list. A mortality rate of 28 deaths per 100 patient years is the rate where ‘harm’ and ‘benefit’ have similar probability for transplanted patients.

**Table 1:** Relative percent of ‘no benefit’, ‘harm’, and ‘benefit’ assuming two-year waiting time

**Mortality: deaths per 100 patient years (Life expectancy)**	**No benefit (%)**^**a**^	**Harm (%)**	**Benefit (%)**	**Ratio harm/benefit**
15 (6.7 years)	26	22	52	0.43
20 (5 years)	33	25	42	0.61
25 (4 years)	39	27	33	0.83
30 (3.3 years)	45	28	27	1.1
35 (2.9 years)	50	29	21	1.4

^a^Does not take into account removal off the wait list. Values were rounded and may not add to 100%.

**Figure 3:**
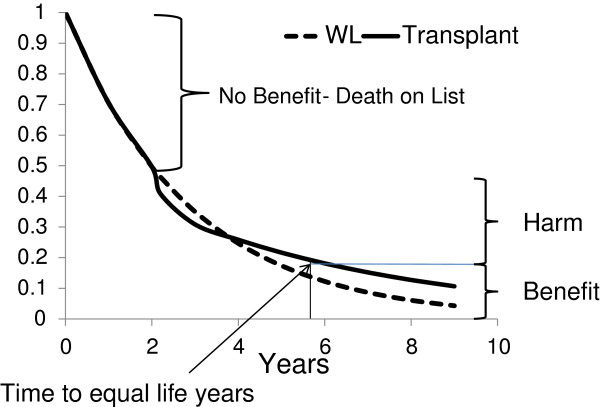
‘No benefit’, ‘benefit’, and ‘harm’ assuming hold wait listed patients are not transplanted and have higher mortality rates.

Assuming that 20% of patients are inactive on the wait list with a 2.2-fold higher mortality rate, those that are actually transplanted have lower baseline mortality rates than the entire wait list reference cohort. Therefore the true reference population wait list also has a lower mortality rate. Table 2 and Figure 3 now show that at a mortality rate of 35 deaths per 100 patient years transplantation is predicted to cause harm (35%) 2.4 times more likely than benefit (15%). The mortality rate where the harm is equivalent to the benefit is now 22 deaths per 100 patient years. This roughly translates to a life expectancy of 4.5 years.

**Table 2:** Relative percent of ‘no benefit’, ‘harm’, and ‘benefit’ assuming a two-year waiting time and 20% of wait list inactive with 2.2-fold higher mortality rate

**Mortality: deaths per 100 patient years (Life expectancy)**	**No benefit (%)**^**a**^	**Harm (%)**	**Benefit (%)**	**Ratio harm/benefit**
15 (6.7 years)	26	29	45	0.63
20 (5 years)	33	32	35	0.93
25 (4 years)	39	34	26	1.3
30 (3.3 years)	45	35	20	1.8
35 (2.9 years)	50	35	15	2.4

^a^Does not take into account removal off the wait list. Values were rounded and may not add to 100%.

Assuming that only 10% of the patients are inactive at any one time changes this equivalency rate to 26 deaths per 100 patient years. Longer waiting time increases the percentage likely to suffer no benefit (die on the list) and reduces the absolute percentages for ‘harm’ and ‘benefit’. However, the ratio of harm to benefit remains unchanged. Table 3 shows that longer wait time increases the percentage that will derive no benefit and diminishes the percentage that shows benefit.

**Table 3:** Relative percent of ‘no benefit’, ‘harm’, and ‘benefit’ assuming a three and four year waiting time and 20% of wait list inactive with 2.2-fold higher mortality rate

**Mortality: deaths per 100 patient years (Life expectancy) Waiting time**	**No benefit (%)**^**a**^	**Harm (%)**	**Benefit (%)**	**Ratio harm/benefit**
15 (6.7 years)				
3 year	36	25	39	0.63
4 year	45	20	33	
20 (5 years)				
3 year	45	27	29	0.93
4 year	55	22	23	
25 (4 years)				
3 year	53	27	20	1.3
4 year	63	22	15	
30 (3.3 years)				
3 year	60	26	14	1.8
4 year	70	19	11	
35 (2.9 years)				
3 year	65	25	11	2.4
4 year	75	18	8	

^a^Does not take into account removal off the wait list. Values were rounded and may not add to 100%.

## Discussion

The model shows that a significant proportion of patients with high mortality rates is not likely to live long enough to receive a transplant and, more importantly, is more likely to be harmed than to benefit. Not transplanting patients with life expectancies < five years (> 20 deaths per 100 patient years) seems to be a reasonable recommendation based on the likelihood of greater harm. Currently, 43% of patients ≥ 65 years of age on the wait list are considered to be high risk (diabetes mellitus or two of the following: ischemic heart disease, cerebral vascular disease, congestive heart failure or peripheral vascular disease) and have an average mortality rate of 22 deaths per 100 patient years [8].

Longer wait times increase the probability of ‘no benefit’ (death on the list) and reduce the likelihood of ‘benefit’. Therefore, centers with long wait times will devote more time and resources evaluating patients that will never be transplanted. This analysis did not take into account removal from the list, which would also be responsible for inefficiency. In the US, 46% of wait listed patients over the age of 65 are never transplanted [11]. Not all die on the list, and some are removed. One strategy is to delay evaluation until patients have sufficient wait time where the likelihood of transplantation is high. Transplant centers with this strategy essentially ‘weed out’ the poor candidates by attrition prior to evaluating for the wait list. In some centers (including our own) with relatively short wait times, the tendency is to be more utilitarian and to test and retest to look for a contraindication to exclude marginal patients. Tests and more tests add to inefficiency, costs, and false expectations. It is conceivable that these different evaluation strategies and center realities are responsible for significant wait list number variations as a proportion of those on dialysis when in fact the actual number and characteristics of patients transplanted are not different.

Unfortunately, accurate unbiased survival calculators are not readily available to clinicians to provide baseline mortality rates. Recent studies on frailty have shown that functional status, in addition to co-morbidities, may be an important determinant of survival [12,13]. Many of the elderly patients that are not referred and have no absolute contraindication to transplantation are likely to be frail and are more likely to be harmed from the transplant surgery and immunosuppression than more robust individuals. Although adding functional measures may improve prognostication, absolute prediction for an individual will never be 100%. Some have argued rather that attempting to ‘quantify the amorphous', we should spend more time discussing uncertainty [14]. Rather than saying a patient has an absolute contraindication as if there is certainty that the patient will be harmed with an intervention, we might suggest the poor candidate is more likely to be harmed than to benefit. Future research is needed not only to improve prognostication, but also in communicating uncertainty to the patient and the community. Ethically, this uncertainty is a more honest construct than arguing a patient is either not a candidate based on ‘do no harm’ (non-maleficence) or not a candidate based on a presumption of limited benefit (utilitarianism). The overall aim is to promote being reasonable but also transparent with the need for ongoing research and reassessment of eligibility decisions [15].

This present analysis uses mortality rates to generate time to equal cumulative life years from the time of transplantation. Wolfe and Gill calculate time to equal survival [6,8]. These are two different time points. Since the survival curve for the transplant cohort is inferior to wait list cohort up to this point, the overall life years (area under the curve) favors the wait list at this point. The time from transplant to the time of equivalent life years is the more critical value.

This study did not calculate net benefits. Projections of net benefit as currently calculated are derived from relatively short follow up times projected over several decades. For instance, the median follow up in the study by Gill was less than three years [16]. The study by Wolfe included patients from 1991 to 1997 [6]. Patient mortality increases exponentially with age (Gompertz’s law) and this needs to be taken into account when projecting patient survival beyond five years especially in older subjects [17,18]. Long-term projected calculations that assume constant mortality overestimate benefit. However, follow up in these studies is likely to be sufficient to calculate time to equal cumulative life years. In addition, prior studies did not take into account hold status of wait listed patients with higher mortality rates [9].

We did not specifically address cohorts with lower mortality rates since the duration of relative risks for transplantation (increased, equal and reduced) might be different and more favorable to transplantation. However, even using this model and assuming many patients on the transplant list have mortality rates of between 5 and 10 deaths per 100 patient years, 65 to 80% would be predicted to benefit with a 10 to 17% probability of harm with transplantation [16]. For pediatric recipients with even lower mortality, the likelihood of benefit would be greater. The probability of ‘harm’ and ‘no benefit’ collectively would approach 10% in this cohort, with 90% deriving benefit.

The model assumes that the period of increased risk remains unchanged and the magnitude of this risk is independent of mortality rate. Further sensitivity analysis on the magnitude of risk or duration of risk for the transplant cohort are problematic since changing one variable will distort survival if adjustments are not made in the remaining variables. There is some evidence that the time of early increased risk is longer and the magnitude of the risk is higher with higher mortality rates. Gill *et al*. recently show that the early peak mortality relative risk (compared to the wait list) in a ‘low risk’ elderly cohort was about 1.7 for a standard donor organ whereas the peak mortality relative risk in a ‘high risk’ elderly cohort was 2.5 [8]. Gill’s study also showed that the relative mortality risk for expanded criteria donors (ECDs) was even higher. Therefore, patients receiving ECDs will also have relatively more harm than benefit compared to non-ECD organs. Taking into account these factors diminishes the projected benefits and increases the likelihood of harm to a greater extent than projected in this theoretical analysis. Therefore, this analysis is overly conservative in favor of transplantation. The model also assumes that the mortality rate is fixed over time and this is a necessary simplification. However, if the mortality rate increases (or decreases if a comorbidity is reversed) the higher (or lower) rate can then be used accordingly.

The analysis does not take quality of life into account, nor does it take into account the pain and suffering related to transplantation surgery and its complications. A contrary argument is that patients should have a choice to take a greater risk of early death for uncertain benefits. This study shows that even in patients with high mortality rates there will always be a small percentage that derives benefit. Some individuals tend to be conservative in their choice of risky interventions and may prefer an existing health state to an uncertain outcome. This paper does not address different individual levels of risk aversion or risk loving. In addition, this is a simulation exercise to consider an alternative perspective to the net benefit model, it is not meant to be definitive. Future registry analysis of large data sets using this approach would be useful. Finally, the concept of ‘harm’ may be strong wording. In some cases early death may be unavoidable and related neither to dialysis nor transplantation.

Tamura, Tan and O’Hare examined decision making in the elderly based on numbers needed to treat to prevent death on the wait list [19]. Unfortunately their model examined age rather than mortality rate, assumed time to equivalent percent survival was the same as time to equivalent life years and assumed that time to equivalent survival did not change with increasing age. Nonetheless, their findings also suggest that the likelihood of transplantation benefiting patients with reduced life expectancies (whether from age or co-morbidity) is low as demonstrated by large numbers needed to be treated to prevent one death.

## Conclusion

In summary this study challenges the premise that all patients who are transplanted will benefit. Rather it proposes that patients with a high inherent mortality rate may be more likely to have their life reduced with a transplant with a smaller probability that there would be a net gain in life. Based on current survival models, patients with mortality rates exceeding 20 to 25 deaths per 100 patient years are at risk of potentially more harm than good.

## Abbreviations

ECD: Expanded criteria donor; MR: Mortality rate.

## Competing interests

The authors declare they have no competing interests.

## Authors’ contributions

BK, conception and design, model development, interpretation of data, manuscript development and final approval. KT, conception and design, interpretation of data, manuscript development and final approval. KW, conception and design, interpretation of data, manuscript development and final approval. All authors read and approved the final manuscript.

## Supplementary Material

Additional file 1: Figure S1 Modeled and observed relative risks of transplant mortality to wait list mortality. **Figure S2.** Modeled and observed relative risks of transplant mortality to wait list mortality, assuming hold patients are not transplanted and have higher mortality rate. Click here for file
