# Supplementary material for: Eligibility for the kidney transplant wait list: a model for conceptualizing patient risk
Source: Transplant Res. 2014 Jan 8;3:2. doi: 10.1186/2047-1440-3-2 (PMC3895784; doi:10.1186/2047-1440-3-2)

Eligibility for the Kidney Transplant Wait List: A Model for Conceptualizing Patient Risk

Kiberd BA et al. 2013

Supporting Information. A description of the assumptions used to develop the computer model and the analytical methodology used to calculate equal life years between patient groups.

The survival model assumed a simple exponential decline and was developed to replicate observed survival curves for wait listed and transplanted cohorts in patients 70+ years.(7) Rao et al found that survival in this cohort was 51% at year 4 for the wait list cohort. Our study finds that a mortality rate of 16.8 deaths per 100 patient years is required to best approximate this observation. This rate is slightly higher that the observed mortality rate in their study of 15.8 deaths per 100 patient years. For the transplanted cohort we assumed 3 periods of differing mortality risk for the transplant cohort relative to the waitlist cohort. In order to replicate the 70+ survival curves by Rao et al, we found that assuming an increased risk period of 0.2 years with a relative risk of 2.26, followed by 0.8 years of equivalent risk and then a reduced risk of 0.44 produced a four year survival of 66% for the transplanted cohort and a time to equal percent survival of 1.6 years. The findings by Rao et al show the time to equal survival is approximately 1.8 years. Therefore we slightly overestimate the benefit of the transplant cohort relative to the wait list cohort in this analysis. On closer inspection (Figure S1) this magnitude of increased risk seems high and the period of equivalent mortality risk seems longer than that published by Rao et al. However, using lower risk mortality hazard ratios or shorter times of equal mortality risk shortens the time to equivalent survival to <1.5 years. Figure S2 shows the impact of eliminating the hold patients (aWL or adjusted wait list reference mortality).

An increase in mortality over time was not included (Gompertz law) as the follow up time was generally <5 years. We assumed that the magnitude of the mortality risks and time for the 3 different risk periods were the same regardless of baseline mortality.

Mortality rates (MR) in deaths per 100 patient years were converted to a survival probability (exp^[-MR*t]) where t is equal to time in years.

To calculate time to equivalent survival in life years and the percentages of no benefit, harm and net benefit the following calculations were made.

1. Increased risk period 0 to 0.2 years.

Wait List (WL) Survival at 0.2 years Swl.2=exp(-MR*0.2)

Transplant (TX) Survival at 0.2 years Stx.2=exp(-MR*HR*0.2)

MR= rate per 100 patient years; HR is increased risk (2.26 or 2.8)

2. Equivalent risk period 0.2 to 1 year

WL Survival at 1 year Swl1= Swl0.2*(exp(-MR*0.8)

TX Survival at 1 year Stx1= Stx0.2* (exp(-MR*0.8)

3. Area under curve for difference 0 to 1 year.

AUCwl0-1 = (1/MR)*(1-exp(-MR*0.2)+Swl.2*(1/MR)*(1-exp(-MR*0.8)

AUCtx0-1 = (1/MR*HR)*(1-exp(-MR*HR*0.2)+Stx.2*(1/MR)*(1-MR*0.8)

4. Time to equivalent life years

Goal seek (Excell™) for t (time after year1 where cumulative life years equal)

(Stx1/MR*rHR)*(1-exp(-MR*rHR*t) + AUCtx0-1=Swl/MR)*(1-exp(-MR*t) + AUCwl0-1

HR now reduced risk value (rHR)

5. No Benefit (2 year wait time)=

1-Survival to transplant on Wait List=1-exp(-MR*2)

6. Benefit (% alive in the transplanted cohort)=

[exp(-MR*2]*[Stx1*exp(-MR*rHR*t)

7. Harm=

1-No benefit-Benefit

8. Adjusted Hazard ratios (aHR)

Assume 20% inactive on list, HR for increased mortality 2.26

MR=0.2*2.26*(aMR)+0.8*(aMR)

aHR=2.26*MR/aMR=2.8 for increased risk time and 0.55 for reduced risk time (vs.0.44)

Figure S1. Modelled and observed relative risks of transplant mortality to wait list mortality


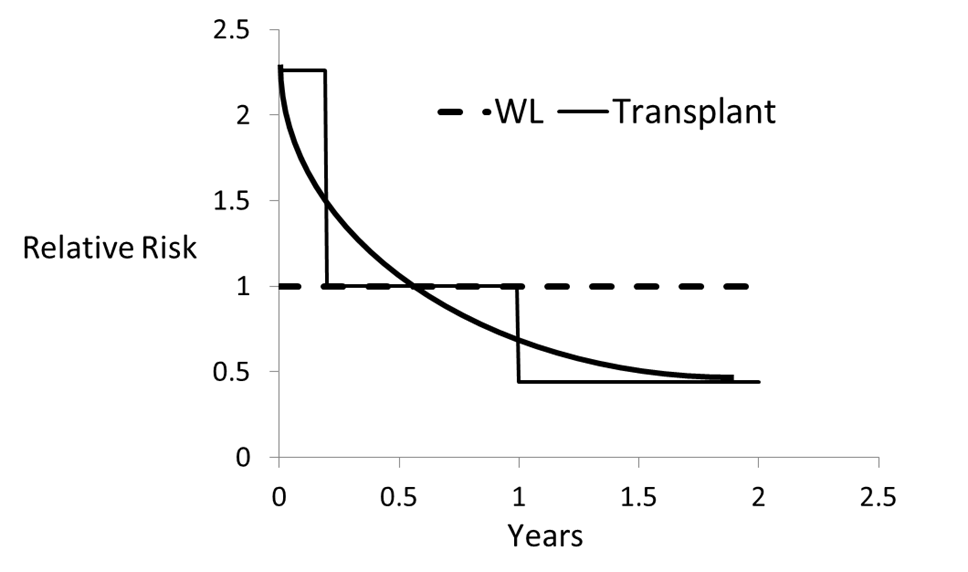


Figure S2. Modelled and observed relative risks of transplant mortality to wait list mortality, assuming hold patients are not transplanted and have higher mortality rate.


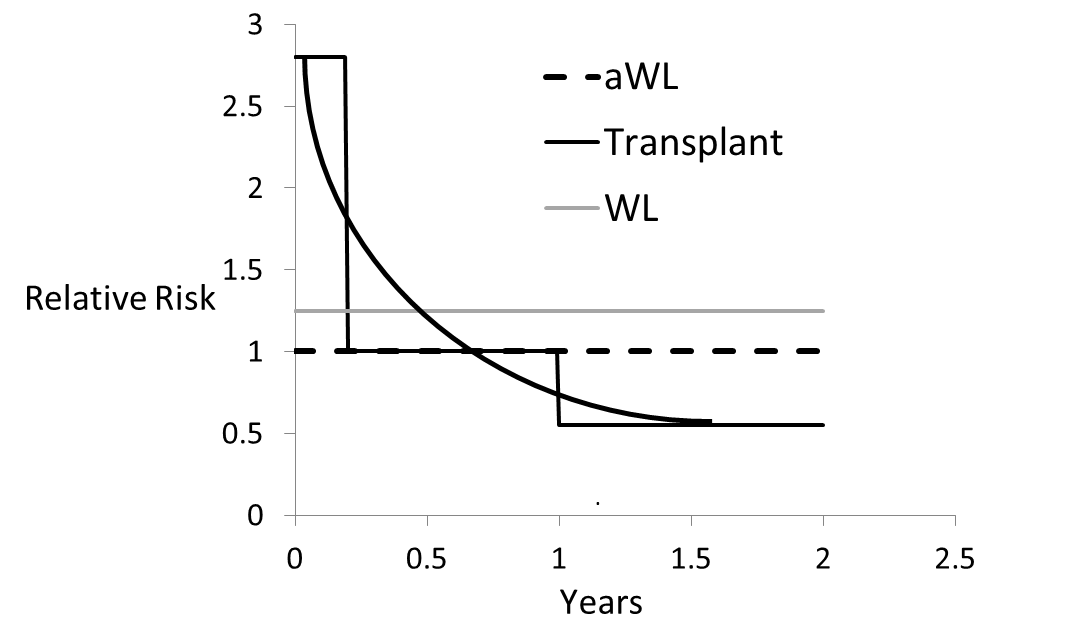

Supplement: Additional file 1: Figure S1 — Modeled and observed relative risks of transplant mortality to wait list mortality. Figure S2. Modeled and observed relative risks of transplant mortality to wait list mortality, assuming hold patients are not transplanted and have higher mortality rate. [file 2047-1440-3-2-S1.docx]
